# Identifying drug-related attributes to personalise antihypertensive agents: the outcome report of patients receiving metoprolol therapy

**DOI:** 10.1186/s12911-021-01739-9

**Published:** 2021-12-30

**Authors:** Chunyu Liu, Jing Xu, Ran Liu, Miye Wang, Yixuan Zhuo, Lan Su, Hongmei Yan, Qing Zhang

**Affiliations:** 1 Pharmacy Department, West China Hospital, Sichuan University, Chengdu, 610041 Sichuan China; 2 Information Technology Department, West China Hospital, Chengdu, 610041 Sichuan China; 3 School of Life Science and Technology, University of Electronic Science and Technology of China, Chengdu, 610061 Sichuan China; 4 Cardiovascular Department, West China Hospital, Chengdu, 610041 Sichuan China

**Keywords:** Hypertension, Personalised medicine, Metoprolol, Drug-related attributes, Data visualization

## Abstract

**Background:**

Currently, numerous antihypertensive drugs from different pharmacological classes are available; however, blood pressure control is achieved in only less than a third of patients treated for hypertension. Moreover, providing optimal and personalised treatment for hypertension is challenging. Therefore, in this study, we propose a ‘drug-related attributes’ sensitive spectrum. This novel concept can assist clinicians in selecting an optimal antihypertensive drug and improve blood pressure control after examining the attributes of a patient.

**Methods:**

We collected clinical data on attributes related to hypertension and its therapy of inpatients from West China Hospital who received metoprolol therapy and constructed the sensitive spectrum using data-visualisation tools.

**Results:**

Our analysis revealed that haematocrit, haemoglobin, serum creatinine, serum cystatin C, serum urea, age, sex, systolic pressure, diastolic pressure, pulse pressure, and heart rate are metoprolol-related attributes.

**Conclusion:**

Our study showed that all metoprolol-related attributes identified are reasonable and helpful in improving the personalisation of metoprolol therapy. The proposed drug-related attributes spectrum can help personalise antihypertensive medication. Moreover, data-visualisation tools can be effectively used to mine the drug-related attributes sensitive spectrum.

## Background

Despite the availability of numerous antihypertensive drugs, hypertension remains a significant public health threat [1], and only 13.8% of the patients with hypertension have their blood pressure (BP) under control worldwide [2]. Although there are several reasons for poor blood pressure control, a likely contributor is the inability to predict antihypertensive drugs to which an individual is most likely to respond. An important element underlying poor responses to the prescribed treatments is the relative lack of predictive approaches to select the most appropriate antihypertensive agent for individual patients [3].

During the past decade, personalised medicine has received considerable attention from clinicians, researchers, drug developers, and regulatory agencies. Personalised medicine includes identifying patients most likely to benefit from and respond to a drug. Perhaps the most effective way to identify the likely responders is by assessing patients' attributes through proteomic, genetic, or other tests. Joint National Committee 7 and 8 of the United States recommended a modest degree of personalisation using patient attributes, such as 12-lead electrocardiography; urinalysis; blood glucose, haematocrit, serum potassium, creatinine, and calcium levels; and fasting lipoprotein profile [4].

Porcher et al. showed that personalising antihypertensive treatment according to predicted benefits and negative effects could eliminate the need for treatment of more than half of the patients and reduce the likelihood of negative effects by more than three times [5]. However, the responses to antihypertensive drugs could be influenced by several environmental or non-genetic factors (e.g., diet, activity, and drug compliance) and their interactions with genetic factors. Therefore, the full realisation of personalised medicine will undoubtedly require a more precise and comprehensive characterisation of individual environmental exposures and measurements across various biological hierarchies [6].

Several studies have identified clinical factors that can be used to guide optimal treatment decisions. For example, according to the findings of the Veterans Affairs Cooperative Studies, age and ancestry influence the response to first-line antihypertensive drugs [3]. Several single nucleotide polymorphism (SNP) association studies have been conducted for hypertension; however, they have been criticised for their poor reproducibility and potential for bias [4]. Given the polygenic nature of hypertension, assessing the efficacy of antihypertensive drugs through genetic testing is complicated in clinical practice [7]. Moreover, the potential of DNA sequences to effectively predict responses to antihypertensive drugs is also doubtful [4]. Considering these challenges, we sought to develop a new, clinically feasible phenotyping method that will allow clinicians to accurately determine an antihypertensive drug for a particular patient.

The hospital information system (HIS), a comprehensive, integrated system used to record and manage all types of clinical information, along with data-visualisation tools, provides a novel tool to accomplish our goal. An excellent HIS should include information on genotypes, phenotypes, biomarkers, polymorphisms, and environmental variations, and thus, provide big data that can be mined for drug-related attributes. In the past decades, several HIS-related applications have been developed, such as prediction of the patient outcomes [8], clinical recommender system [9], detection of drug–drug interactions [10], and risk prediction [11]. Data-visualisation techniques can not only extract useful information from big data (i.e., data mining) but also intuitively display the results obtained [12]. Moreover, data visualisation can present the data mining results in a visual context and thus provide various options to understand the significance of key health indicators. With an effective visualisation approach, patterns, trends, and associations can be clearly extracted and communicated, which may otherwise be overlooked, undetected, or hidden deep in big data [13]. Moreover, data-visualisation tools can facilitate a better understanding of the data by both patients and physicians and improve the quality of treatment.

Metoprolol is a Food and Drug Administration (FDA)-approved drug for hypertension, and it functions as a beta-blocker to control high BP. This drug acts by reversing the effect of certain stress hormones, such as renin, angiotensin, and adrenaline in the body. Although it does not cure high BP, it helps decrease the heart rate, BP level, and heart workload. Moreover, metoprolol has several indications on its official label and lowering of BP in response to metoprolol therapy widely differs among individuals [14]. Considering these facts, hypertension guidelines worldwide typically include metoprolol as a second-line antihypertensive [4]. Moreover, clinicians are often puzzled and hesitant to prescribe metoprolol to a patient. We hypothesized that developing a model for effective selection of the drug could be helpful in a clinical setup.

In this study, we used data-visualisation tools to identify metoprolol-related characteristics from the clinical attributes of patients with hypertension to facilitate the personalised application of metoprolol in clinical settings. The study provides evidence that the use of drug-related attributes for prescribing a personalised medication is practically feasible.

## Methods

### Experiment design

In our study, we focused on patients with hypertension receiving metoprolol monotherapy. Generally, an object or the status of an object at a particular time can be systematically and accurately described using various attributes of the object itself, which also applies to patients. We were inspired by how an antibacterial spectrum of an antibiotic or drug is presented. We can list all attributes of a patient into a set where each item is sensitive to the targeted drug’s effectiveness or a specified indication. The sensitive item can be any patient attribute, such as symptoms, laboratory results, clinical treatments, genetic polymorphisms, phenotypes, biomarkers, and environmental variations. All factors discussed above are attributes related to the targeted drug effect or decision. We termed these attributes as ‘drug-related attributes’, indicating that they are key and sensitive to the drug's efficacy and can help clinicians select the appropriate drug. These drug-related attributes in the set are similar to those in the antibacterial spectrum; thus, we termed this set as ‘drug-related attributes spectrum’. A physician can easily choose a personalised drug by matching the patient’s attributes with those in the drug-related attributes spectrum, and the effectiveness of the selected drugs can be ensured. Of course, not all attributes influence the outcomes, and not all of them have similar effects. Only a few attributes are significantly related or sensitive to the outcomes of patients with hypertension receiving metoprolol monotherapy, and we classified them as ‘metoprolol-related attributes’. Although there were numerous algorithms to categorise these attributes, such as rough set and deep learning, in this study, we categorised the attributes based on data visualisation, as this method is simple and straightforward. Moreover, only a few studies in this field have used this method.

**Fig. 1:**
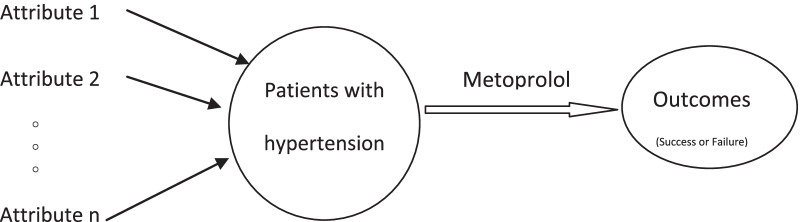
Overview of the experiment method

The logic model for our experiment is briefly elucidated in Fig. 1.

### Data source and population

For this study, patients were not selected but extracted from a real-world database, thus reducing the risk of selection bias. In China, a systematic record of hypertensive outpatients is not available. Therefore, we collected inpatient data from the HIS of West China Hospital with more than 6000 inpatient beds and systematic HIS records dating back to more than 15 years (this was the major data source of our study). This study does not involve human or animal participants. All data were de-identified before being collected to comply with ethics requirements.

The data population included all inpatients who received metoprolol monotherapy and registered in the data source from Jan 2012 to Dec 2018 (the reference period), and whose diagnosis had the term ‘hypertension' mentioned on their discharge reports. Antihypertensive treatment in outpatient situations is common, but the data of hypertensive outpatients were not traceable in our hospital. However, our hospital had sufficient inpatients with hypertension to satisfy our research requirements.

As our targeted population comprised inpatients, the diagnosis of hypertension had been made before the initiation of metoprolol monotherapy. All BP measurements were performed in wards by trained nurses following physicians' instructions. Considering that the protocol for BP measurement and criteria for hypertension diagnosis have not changed over the past 10 years, the BP measurement and diagnosis data were compiled as per the criteria mentioned in 'Chinese Guidelines for Prevention and Treatment of Hypertension in 2018'.

Based on the pharmacokinetics and pharmacodynamics of metoprolol in the official label, we chose the initiation day of antihypertensive treatment as a reference point. The average BP of five continuous days immediately after the reference point was used as outcome BP to determine whether the therapy was successful. Patients who showed a systolic pressure (SB) of less than 140 mmHg and a diastolic pressure of less than 90 mmHg comprised the successful group. On the contrary, patients whose BP was outside the above-mentioned range comprised the unsuccessful group. For the attributes of the patients that were not examined by the laboratory staff every day, we adopted the values measured within three days of the reference point.

### Data extraction

We looked for three types of data for our study: data of patients who received metoprolol monotherapy, attributes of the targeted patients that can delineate the patients' status just before metoprolol administration, and 5-day BP outcome of the targeted patients just after initiating metoprolol treatment.

Based on clinical reports [15–24] and expert knowledge, we first listed approximately 50 features related to hypertension and its therapy. Only 19 of these features were available in the HIS of West China Hospital. Therefore, we finally selected the following targeted attributes: haematocrit (HCT), haemoglobin (HB), serum creatinine(Cr), serum cystatin C (Cys), serum urea, serum uric acid (UA), cholesterol (TC), low-density lipoprotein cholesterol (LDL), high-density lipoprotein cholesterol (HDL), potassium, sodium, and fasting blood glucose (FPG) levels; platelet (PLT) count; age; sex; SB; diastolic pressure (DB); pulse pressure (PP); and heart rate (HR). We obtained the values of the targeted attributes measured for up to three days before initiating metoprolol treatment, as some tests were not performed every day.

### Data cleaning

From the population data, patients who were taking antihypertensive agents other than metoprolol and those whose metoprolol dose was not based on the label were excluded. International metrological units were applied to all 19 targeted attributes. Any 'null' or 'blank' values were considered invalid, and the associated records were removed from the data.

### Data normalisation

For a better comparison of the attributes, values of all 19 attributes were converted to a standard range by Min–Max normalisation [25] using the following formula:

$${\text{Normalised value}} = ({\text{original value}} - {\text{minimised value}})/({\text{maximised value}} - {\text{minimised value}}).$$

The clinical references of all the targeted attributes were normalised, as shown in Table 1. After normalisation, the values of each attribute ranged between 0 and 1.

**Table 1:** Normalisation of clinical references of the 19 targeted attributes

Attribute	Attribute max	Attribute min	Reference range	Normalised reference
HCT	0.54	0.13	0.35–0.45	0.537–0.780
HB	185	44	110–150	0.468–0.752
PLT	494	2	100–300	0.200–0.606
Cr	1161	4	44–132	0.035–0.111
Urea	34	0.86	3.1–7.1	0.070–0.188
UA	743	13.4	150–420	0.187–0.557
Cys	8.58	0.08	0.59–1.03	0.060–0.112
TC	9.62	0.21	3.1–5.7	0.307–0.583
LDL	6.54	0.01	1.5–3.36	0.228–0.513
HDL	2.85	0.08	0.8–1.6	0.260–0.549
K^+^	6.17	2.42	3.5–5.5	0.288–0.821
Na^+^	172.2	124	135–145	0.228–0.436
FPG	22.9	0.76	3.9–6.1	0.142–0.241
Age	104	7		
Sex	2 (Female)	1 (Male)	1,2	[0,1]
SBP	263	67	90–140	0.117–0.372
DBP	168	18	60–60	0.280–0.480
PP	128.5	10	20–60	0.084–0.422
HR	172	20	60–100	0.263–0.526

HCT: haematocrit (L/L), HB: haemoglobin (g/L), Cr: serum creatinine (μmol/L), Cys: serum cystatin C (mg/L), Urea: serum urea (mmol/L), UA: serum uric acid (μmol/L), TC: cholesterol (mmol/L), LDL: low-density lipoprotein cholesterol (mmol/L), HDL: high-density lipoprotein cholesterol (mmol/L); K^+^: potassium (mmol/L); Na^+^: sodium (mmol/L); FPG: fasting blood glucose (mmol/L); PLT: platelet count (109/L); Age (years); Sex (male/female); SB: systolic pressure (mmHg); DB: diastolic pressure (mmHg); PP: pulse pressure (mmHg); HR: heart rate (per min)

### Data visualisation

The data were visualised using the Matplotlib tool, Python 3.4 (downloaded from https://www.python.org/downloads/release/python-340/). The attributes were plotted on the X-axis, and the normalised values of each attribute were on the Y-axis. The number or frequency of patients with x attribute at y value is represented by the corresponding colour on the colour bar. The brighter the colour, the higher the number or frequency.

We first visualised the overall data of all patients on metoprolol monotherapy, including the successful and unsuccessful groups. We then visualised the difference in data between the groups to identify the attributes that could have had a significant effect on metoprolol treatment outcomes. Finally, we used the average values of attributes to verify our selection.

**Fig. 2:**
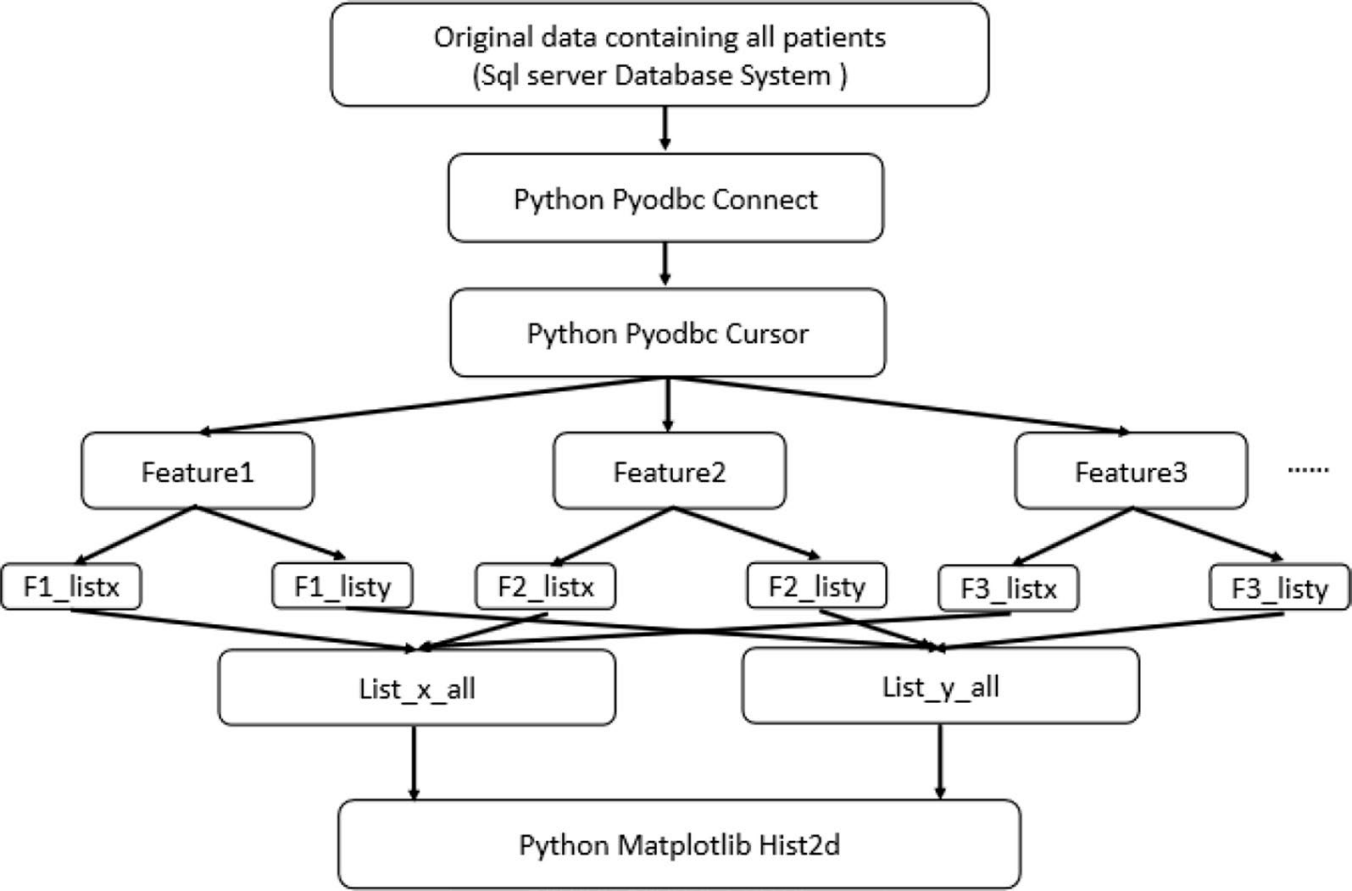
Flowchart of realisation of our data-visualisation tool. Pyodbc is a module that is responsible for operating the database. Fx_listx represents the value list of feature x, Fx_listy represents the given-label list of feature x

Figure 2 shows how our data-visualisation tool will be realised.

## Results

**Fig. 3:**
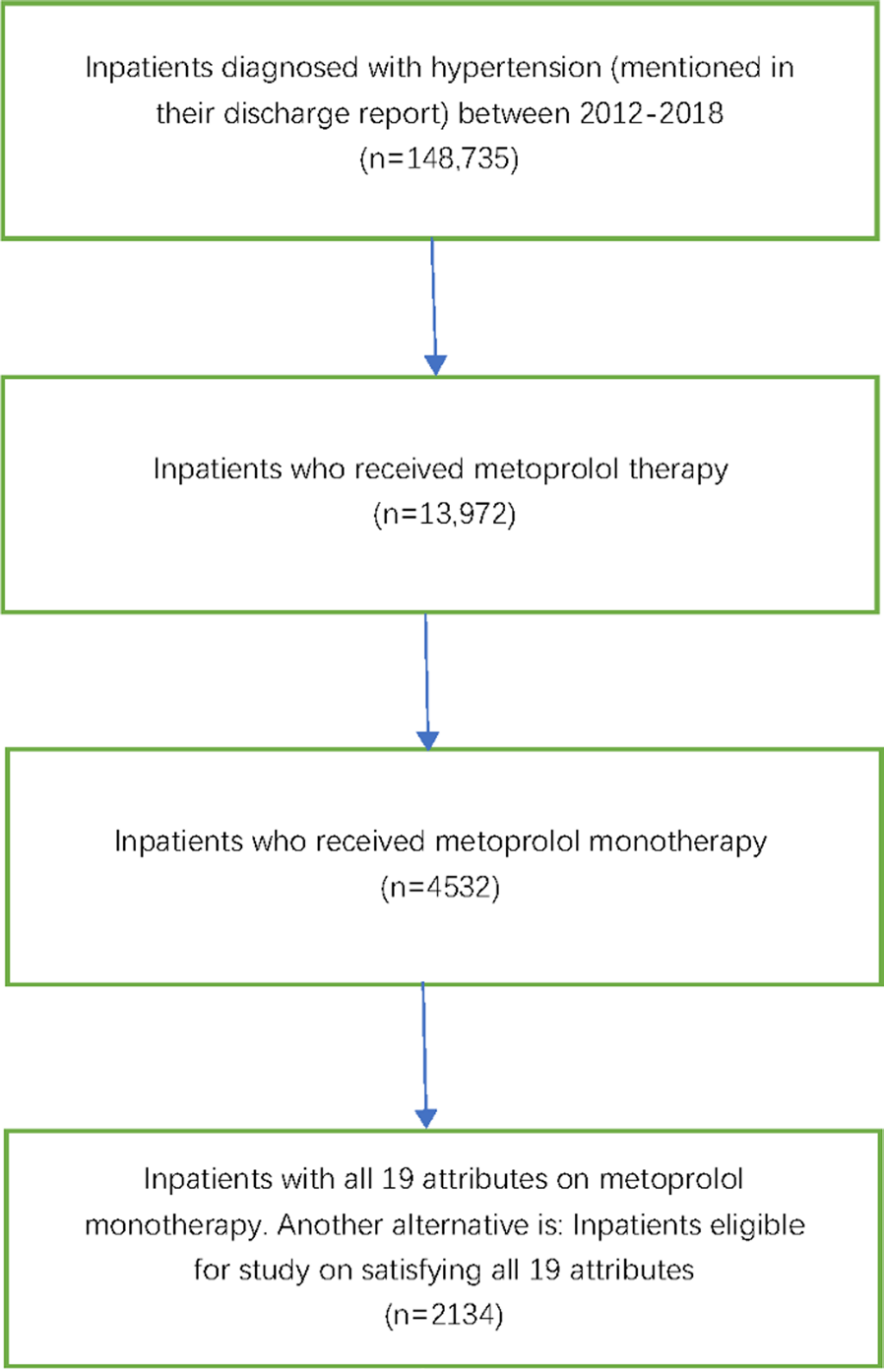
Flowchart of patient selection. The hypertension diagnosis complied with the 'Chinese Guidelines for Prevention and Treatment of Hypertension in 2018'

During the reference period, 148,735 patients with hypertension were admitted to West China Hospital. From these patients, we extracted 13,972 patients who received metoprolol, including 4532 patients receiving metoprolol monotherapy. After data cleaning, only 2134 patients satisfied our study requirements. Figure 3 shows a flowchart of patient selection, including the inclusion and exclusion criteria.

**Fig. 4:**
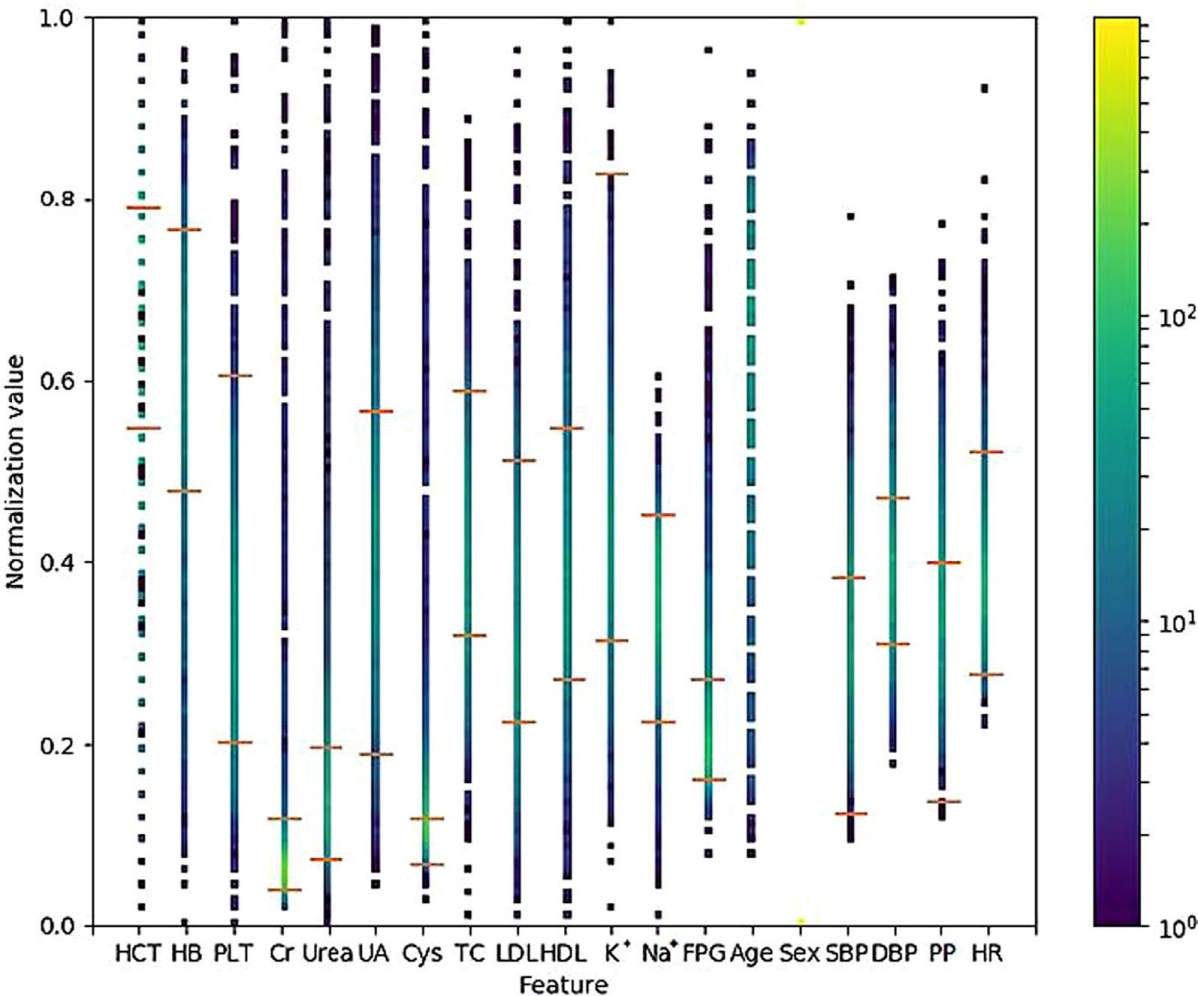
Attribute distribution of all patients who received metoprolol. The point colour of each dotted line represents the normalised value of the targeted attribute for each patient in the successful and unsuccessful groups. The two red horizontal bars on the dotted lines represent the lowest and highest values of the normalised reference values of the attributes. HCT: haematocrit; HB: haemoglobin; Cr: serum creatinine; Urea: serum urea, UA: serum uric acid; Cys: serum cystatin C; TC: cholesterol; LDL: low-density lipoprotein cholesterol; HDL: high-density lipoprotein cholesterol; K^+^: potassium; Na^+^: sodium; FPG: fasting blood glucose; PLT: platelet count; SBP: systolic pressure; DBP: diastolic pressure; PP: pulse pressure; HR: heart rate. The colour shade on the right is the Heatmap of the figure. The normalised attribute values are shown in different colour codes. The low values (at the bottom) are shown in purple, and the high values are shown in yellow (at the top)

**Fig. 5:**
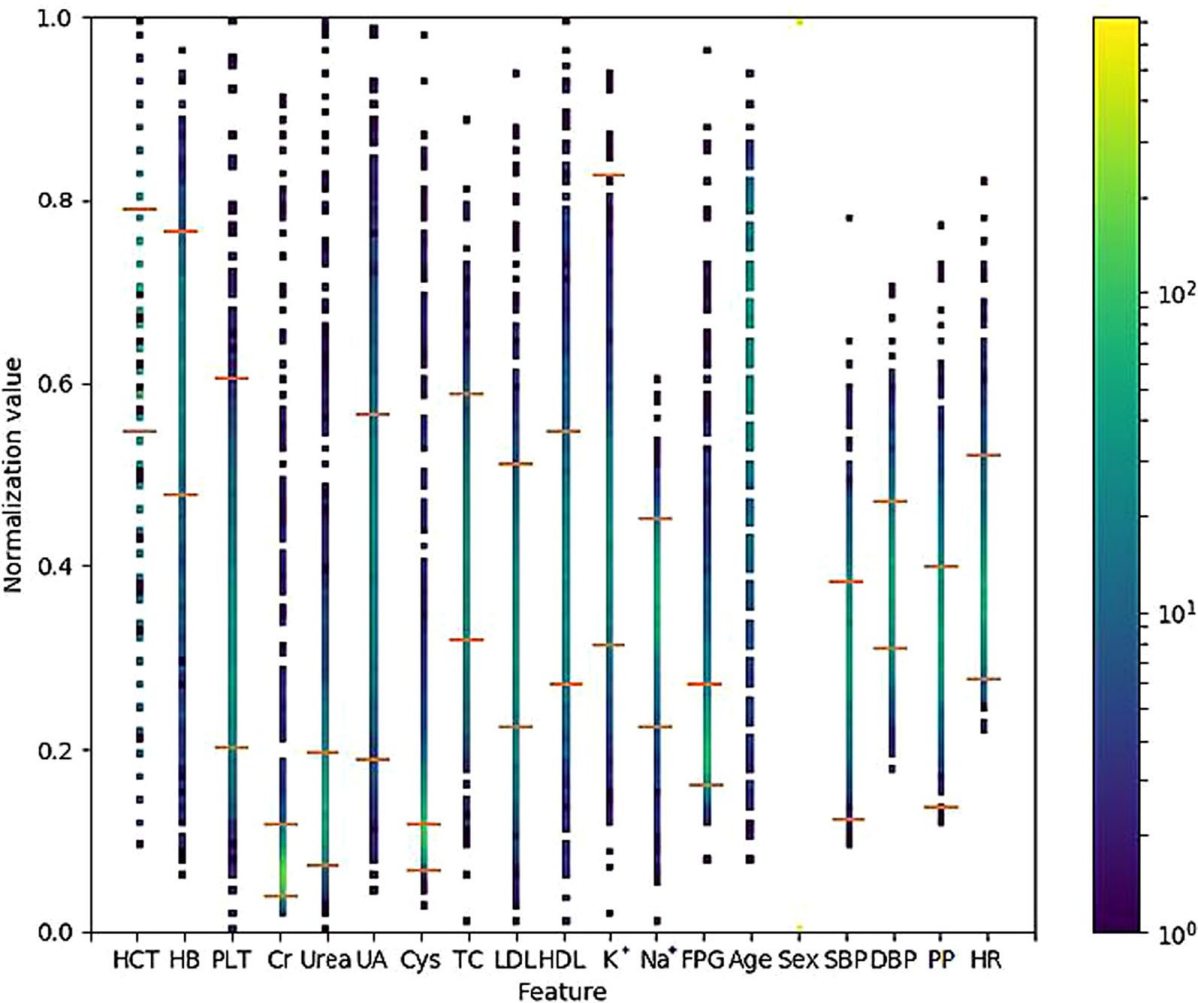
Attribute distribution of the successful group. The point on each dotted line represents the normalised value of the targeted attribute of each patient in the successful and unsuccessful group. The two red horizontal bars on the dotted lines represent normalised reference values of the attributes. HCT: haematocrit; HB: haemoglobin; Cr: serum creatinine; Urea: serum urea, UA: serum uric acid; Cys: serum cystatin C; TC: cholesterol; LDL: low-density lipoprotein cholesterol; HDL: high-density lipoprotein cholesterol; K^+^: potassium; Na^+^: sodium; FPG: fasting blood glucose; PLT: platelet count; SBP: systolic pressure; DBP: diastolic pressure; PP: pulse pressure; HR: heart rate. The colour shade on the right is the Heatmap of the figure. The normalised attribute values are shown in different colour codes. The low values (at the bottom) are shown in purple, and the high values are shown in yellow (at the top)

**Fig. 6:**
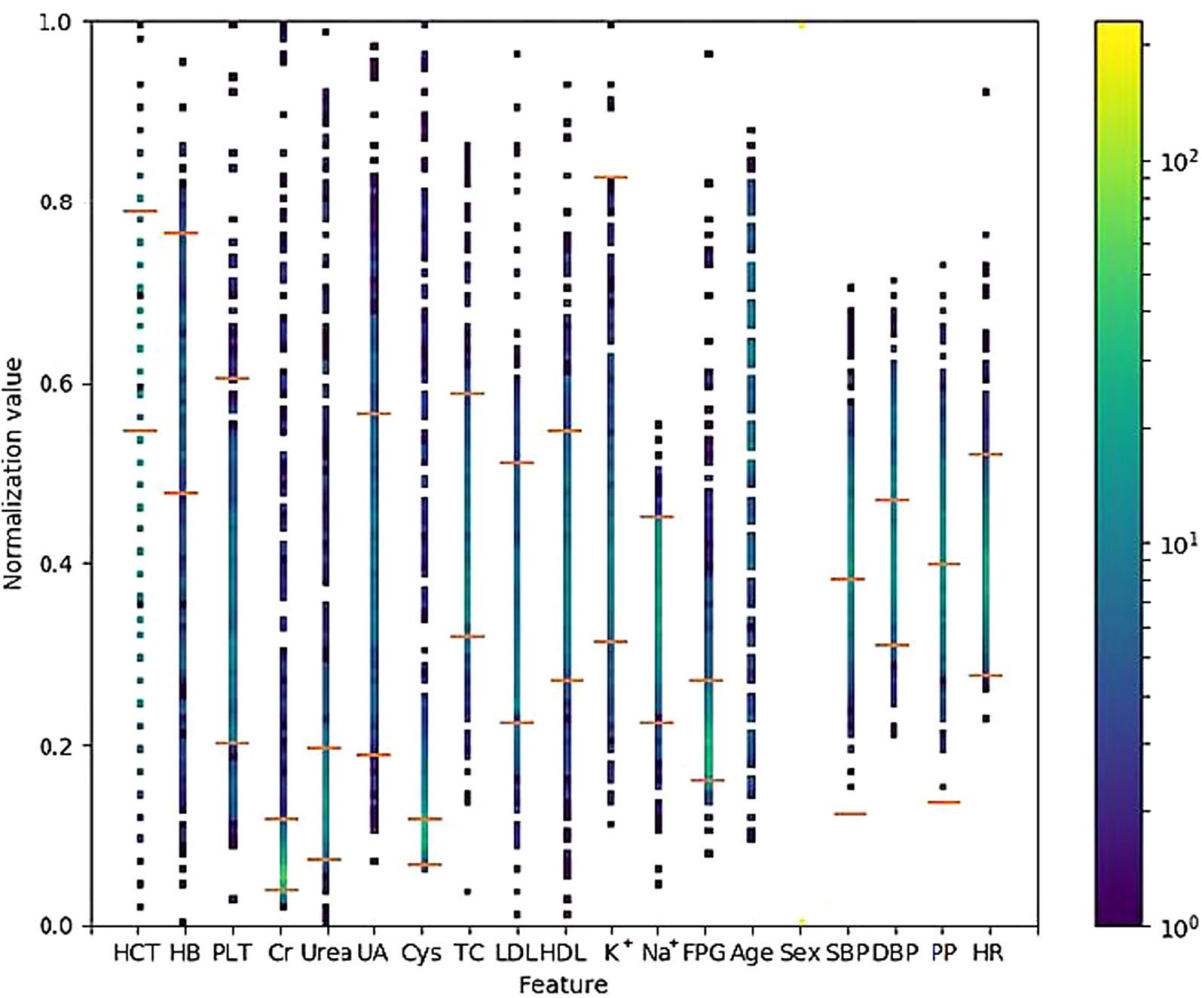
Attribute distribution of the unsuccessful group. The point colour on each dotted line represents the normalised value of the of the targeted attribute of each patient in the successful and unsuccessful groups. The two red horizontal bars on the dotted lines represent normalised reference values of the attributes. HCT: haematocrit; HB: haemoglobin; Cr: serum creatinine; Urea: serum urea, UA: serum uric acid; Cys: serum cystatin C; TC: cholesterol; LDL: low-density lipoprotein cholesterol; HDL: high-density lipoprotein cholesterol; K^+^: potassium; Na^+^: sodium; FPG: fasting blood glucose; PLT: platelet count; SBP: systolic pressure; DBP: diastolic pressure; PP: pulse pressure; HR: heart rate. The colour bar on the right represents a Heatmap of the data. The normalised attribute values are shown in different colour codes. The low values (at the bottom) are shown in purple, and the high values are shown in yellow (at the top)

We plotted the patient attribute data using our data-visualisation tools. The distribution of attributes among all patients is presented in Fig. 4, and that in the successful and unsuccessful groups are presented in Figs. 5 and 6, respectively.

Figures 4, 5, and 6 provide an overview of the distribution of each attribute for all patients: the successful group, unsuccessful group, and the highest and lowest values of each attribute. Like the spectrum of a chemical compound, which reflects the characteristics of the compound, these figures represent the 'attributes spectrum' and elucidate the status of the metoprolol monotherapy group. Furthermore, with an increase in data points, the spectrum becomes more stable. The analysis suggests that if a patient has all 19 targeted attribute values, a physician can easily dot out the location in the ‘attributes spectrum’ and obtain a good profile of the patient's status before selecting an antihypertensive agent. Although we cannot clarify the important factors related to metoprolol, we can identify the 'drug-related attributes' spectrum from the 'total attributes' spectrum.

**Fig. 7:**
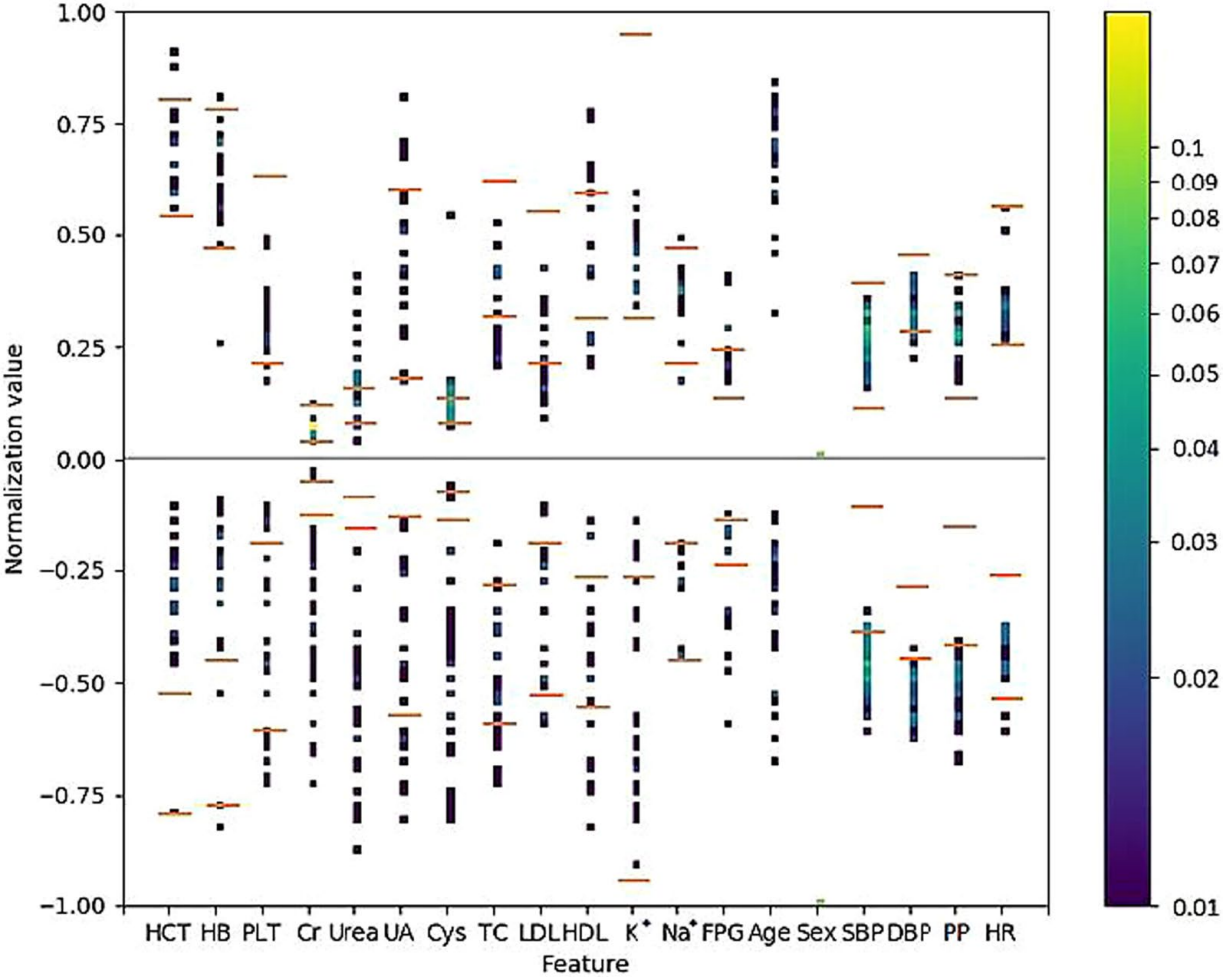
Differences in attribute distribution between the successful and unsuccessful groups. The point colour on each dotted line represents the normalised value of the targeted attribute of each patient in the successful and unsuccessful groups. The two red horizontal bars on the dotted lines represent normalised reference values of the attributes. HCT: haematocrit; HB: haemoglobin; Cr: serum creatinine; Urea: serum urea, UA: serum uric acid; Cys: serum cystatin C; TC: cholesterol; LDL: low-density lipoprotein cholesterol; HDL: high-density lipoprotein cholesterol; K^+^: potassium; Na^+^: sodium; FPG: fasting blood glucose; PLT: platelet count; SBP: systolic pressure; DBP: diastolic pressure; PP: pulse pressure; HR: heart rate. The colour bar on the right represents the Heatmap of the data. The normalised attribute values are shown in different colour codes. The low values (at the bottom) are shown in purple, and the high values are shown in yellow (at the top)

Figure 7 shows the differences in attribute distribution between the successful and unsuccessful groups. The differences were calculated by subtracting the frequencies of the unsuccessful group from those of the successful group.

In Fig. 7 (summarized in Table 2), all attribute-value dot lines fall into three categories. (1) Symmetrical or nearly symmetrical (Is asymmetrical?’ ‘No’; Has a breakpoint?’ ‘/’ (nosense); Table 2): in this category, the distribution of the normalised value of patient frequency for a targeted attribute in the successful and unsuccessful groups are symmetrical or nearly symmetrical. For example, UA and HDL attributes in the successful and unsuccessful groups have a similar distribution at any point. In other words, these attributes cannot distinguish the response to metoprolol between the two groups; therefore, they cannot help in personalising medicine. (2) Unsymmetrical with a breakpoint (‘Is asymmetrical?’ ‘Yes’; Has a breakpoint?’ ‘Yes’; Table 2): in this category, the distribution of the normalised value of patient frequency for a targeted attribute in the successful and unsuccessful groups are almost unsymmetrical, and at a certain point of these attribute lines, the successful group dominates one side, wherein the unsuccessful group dominates the other side (referred as to ‘breakpoint’). In this situation, the value of an attribute can indicate the group that will benefit. For example, HCT, Cr, and HB. These attributes can be considered as metoprolol-related attributes and strongly aid in personalising medicine. (3) Unsymmetrical and without a breakpoint (‘Is asymmetrical?’ ‘Yes’; Has a breakpoint?’ ‘No’; Table 2): the distribution of the attribute value dotted line in the successful and unsuccessful groups is unsymmetrical. In all such attributes, the values between the successful and unsuccessful groups are interlinked and random. In other words, selecting a break point in one group might not define the opposite group dots—for example, PLT and TC. However, mining more data for these drug-related attributes might help identify the break point.

**Table 2:** The attribute distribution between the successful and unsuccessful groups: summarisation of data shown in Fig. 7

Attribute	Is asymmetrical?	Has a breakpoint?	Breakpoint estimate	Important^a^
HCT	Yes	Yes	0.58	Yes
HB	Yes	Yes	0.52	Yes
PLT	Yes	No		No
Cr	Yes	Yes	0.10	Yes
Urea	Yes	Yes	0.24	Yes
UA	No	/		No
Cys	Yes	Yes	0.18	Yes
TC	Yes	No		No
LDL	Yes	No		No
HDL	No	/		No
K^+^	Yes	No		No
Na^+^	No	/		No
FPG	No	/		No
Age	Yes	Yes	0.58	Yes
Sex	Yes	Yes		Yes
SBP	Yes	Yes	0.35	Yes
DBP	Yes	Yes	0.42	Yes
DP	Yes	Yes	0.35	Yes
HR	Yes	Yes	0.37	Yes

^a^Criteria of ‘Important’: The attribute whose value dotted line is unsymmetrical with a breakpoint

To support our above findings, we calculated the average of the 19 attribute values from the total of the successful and unsuccessful groups. The differences between the groups and their associated key results are shown in Table 3.

**Table 3:** Average attribute values and the difference between the successful and unsuccessful groups

Attributes	Inpatients group	Successful group value	Unsuccessful group value	Value difference	Important^a^
HCT	0.5758	0.5242	0.5949	− 0.0707	Yes
HB	0.5381	0.4881	0.5566	− 0.0685	Yes
PLT	0.3462	0.3620	0.3404	0.0216	No
Cr	0.1307	0.2005	0.1050	0.0955	Yes
Urea	0.2254	0.2897	0.2017	0.0881	Yes
UA	0.4491	0.4559	0.4466	0.0093	No
Cys	0.1979	0.2732	0.1701	0.1030	Yes
TC	0.4102	0.4314	0.4025	0.0289	No
LDL	0.3362	0.3545	0.3295	0.0250	No
HDL	0.4148	0.4154	0.4146	0.0008	No
K^+^	0.4352	0.4428	0.4324	0.0104	No
Na^+^	0.3469	0.3430	0.3483	− 0.0053	No
FPG	0.2578	0.2601	0.2570	0.0032	No
Age	0.5746	0.5286	0.5916	− 0.0630	Yes
Sex	0.4362	0.5044	0.4110	0.0934	Yes
SBP	0.3432	0.4109	0.3182	0.0927	Yes
DBP	0.4028	0.4447	0.3874	0.0573	Yes
DP	0.3697	0.4161	0.3527	0.0634	Yes
HR	0.3999	0.4134	0.3949	0.0185	Yes

^a^Criteria of 'Important': The attribute whose value dotted line is unsymmetrical with a breakpoint

## Discussion

Our analysis revealed that HCT, HB, Cr, Urea, Cys, age, sex, SBP, DBP, PP, and HR are important and sensitive attributes (Table 2). For any attribute to be considered important or sensitive, its absolute value difference between the successful and unsuccessful groups should be higher than that of an unimportant attribute. The higher the absolute value difference, the greater is the significance. The data presented in Table 3 further support our results of data visualisation and analysis.

Interestingly, we found that the unsuccessful group was strongly associated with the lower HCT and HB levels, indicating that HCT and HB are sensitive attributes for metoprolol treatment. A study involving 5616 individuals over 10 years has shown that both higher HCT levels (even those within the normal range at baseline) and an increase in HCT level over time are associated with the incidence of hypertension, independent of other related risk factors [26]. The authors concluded that the HCT level has an important role in the development of hypertension. Therefore, we inferred that the antihypertensive mechanism of metoprolol involves the reduction in the level of HCT, i.e., if a patient has a high HCT level, reduction in that level by metoprolol, and vice versa, can be beneficial. Metoprolol also has a higher efficiency in patients with a high HB level than that in patients with a low HB level; this might be because (1) the HCT level is positively correlated with hypertension, (2) blood viscosity varies directly with the HCT level, and (3) there is a strong association between blood viscosity and arterial pressure [27]. As HB is an iron-containing metalloprotein in the red blood cells, it is understandable that the HCT and HB levels have similar predictive trends. However, the HCT and HB levels are often neglected by clinicians when prescribing antihypertensive agents.

Our results showed that the successful group is significantly associated with lower levels of Cr, urea, and Cys. However, the levels of TC, LDL, HDL, and FPG are not included in our drug-related attributes spectrum. The Cr, urea, and Cys levels are commonly used to determine renal function [28]. Viazzi et al. have shown that the kidneys play an important role in the pathogenesis of hypertension [29]. These studies suggest that the deterioration of renal function is an indicator of the development of hypertension. However, our study shows that metoprolol monotherapy might not be sufficient for controlling hypertension in patients with renal dysfunction. The TC, LDL, HDL, and FPG levels may not be accurate indicators of hypertension deterioration; furthermore, our data show that they are not essential factors for indicating the effectiveness of metoprolol.

In a hypertension treatment study, the Hypertension Care Computing Project of the Department of Health and Social Security showed improved survival in hypertensive men treated with beta-blockers; however, this effect was not observed in women [30]. Consistent with these findings, our results reveal sex as a sensitive attribute.

Quarterman et al. reported that the effect of age on the pharmacokinetics of metoprolol and its metabolites is less pronounced than that observed for other drugs [31]. Natale et al. indicated that metoprolol is more likely to benefit older patients [32]. Our data showed that older patients with hypertension exhibit a favourable response to metoprolol. However, more studies are needed to corroborate this conclusion.

The inclusion of SBP, DBP, PP, and HR in the metoprolol-related attribute spectrum is not unexpected and does not need comprehensive discussion. However, it indicates that metoprolol monotherapy may not be sufficient if a patient has multiple deteriorated attributes.

Our results revealed an obvious difference between metoprolol-related attributes and risk factors that clinicians use extensively to diagnose hypertension. Therefore, we suggest that diagnosis must be based on a logic different from that used for drug selection. By matching metoprolol-related attributes, clinicians can focus on the sensitive attributes to improve metoprolol personalisation. Different antihypertensive agents have different pharmacodynamics; therefore, they could have diverse drug-related attributes spectrum. We strongly suggest that drug-related attributes be established to aid in the personalisation of therapeutic drugs. The need for personalisation of hypertension therapy has been documented in the Joint National Committee (JNC) Reports [33, 34]. As antihypertensive drug responses may be influenced by various genetic and environmental factors and their interactions, complete realisation of personalised medicine will undoubtedly require more precise and comprehensive characterisation of individual environmental exposures and measurements from multiple levels across biological hierarchy [6]. To the best of our knowledge, this is the first time a novel, meaningful personalisation method based on drug-related, specific, testable attributes of patients has been proposed to establish drug-related attributes sensitive spectrum using data visualisation. Our metoprolol-related attributes provide a good understanding of this proposal.

In conclusion, our study suggests that HCT, HB, Cr, Urea, Cys, age, sex, SBP, DBP, and PP can be considered metoprolol-related attributes when clinicians personalise antihypertensive agents.

In Table 3, all 'Yes' items, except HR, had a higher absolute value of the difference (> 0.05), and the other items had a lower absolute value of the difference (< 0.05). These results supported the data visualisation results and analysis.

First, although our study was limited by the population studied, which involved inpatients only, and had limited applicability for the use of metoprolol specifically as an antihypertensive agent, our HIS had data of 148,735 patients with hypertension. Among these patients, 2134 satisfied our requirement of having all 19 attributes available. Second, considering that our focus was on the practical application of the personalisation method, some important attributes, such as biomarkers, genetic polymorphism, and phenotypes, which are not extensively used, were not analysed in this study. The metoprolol-related attributes spectrum needs further expansion and improvement by incorporating these important attributes. Nevertheless, metoprolol-related attributes identified in this study could be a good reference for clinicians to personalise medicine. The use of artificial intelligence models based on our drug-related attributes spectrum could further improve this process.

## Conclusions

We established a drug-related attributes sensitive spectrum and explored its practicability for the personalisation of medication. Our study shows that the drug-related attributes spectrum can help clinicians personalise therapy. Further research is needed to ensure each drug-related attribute complements each drug or therapeutic regiment. As different attributes have different effects on patient status, quantitative algorithms based on drug-related attributes can be constructed using artificial intelligence to derive practical applications based on our idea.

## Data Availability

The data that support the findings of this study are available from the HIS of West China Hospital, upon approved request. Contact the corresponding authors.

## Abbreviations

BP: Blood pressure
Cr: Serum creatinine
CYS: Serum cystatin C
DB: Diastolic blood pressure
FPG: Fasting blood glucose
HB: Haemoglobin
HCT: Haematocrit
HDL: High-density lipoprotein cholesterol
HIS: Health information system
HR: Heart rate
LDL: Low-density lipoprotein cholesterol
PLT: Platelet count
PP: Pulse pressure
SB: Systolic blood pressure
SNP: Single nucleotide polymorphism
TC: Cholesterol
UA: Serum uric acid
